# Finite Element Analysis of Different Double-Plate Angles in the Treatment of the Femoral Shaft Nonunion with No Cortical Support opposite the Primary Lateral Plate

**DOI:** 10.1155/2018/3267107

**Published:** 2018-07-31

**Authors:** Hao Zhang, Jiantao Li, Jianfeng Zhou, Lianting Li, Ming Hao, Kun Wang, Gaoxiang Xu, Chen Li, Wei Zhang, Peifu Tang

**Affiliations:** ^1^Department of Orthopaedics, Chinese PLA General Hospital, No. 28 Fuxing Road, Beijing 100853, China; ^2^Department of Emergency, Chinese PLA General Hospital, No. 28 Fuxing Road, Beijing 100853, China; ^3^Department of Orthopaedics, The Third People's Hospital of Qingdao, No. 29 Yongping Road, Qingdao 266041, China; ^4^Department of Orthopaedics, Tianjin Hospital, No. 406 Jiefang Road, Tianjin 300211, China

## Abstract

**Objectives:**

We evaluated the biomechanical outcome of different plate fixation strategies (the single plate construct, 45° double-plate construct, 90° double-plate construct, 135° double-plate construct, and 180° double-plate construct) used for the fixation of the femoral shaft nonunion with no cortical support opposite the primary lateral plate. This may help surgeons choose the optimal therapy to the femoral shaft nonunion.

**Methods:**

The femoral shaft nonunion with no medial support and the models of lateral plate and medial plate was constructed in 3-matic software and UG-NX software, respectively. We then assembled the single plate and different double plates to the fracture model separately to form the fixation models. After meshing the models' elements, we used the ABAQUS software to perform the finite element analysis. Values of the von Mises Stress (VMS) distribution of the implant, peak VMS, and model displacement and deformation were used to capture the mechanical factors in this study.

**Results:**

Our results indicated that the peak von Mises Stress (VMS) of the lateral plate was concentrated in middle surface of the lateral plate near the fragment of each group. The peak VMS was 5201.0 MPa (the single-plate construct), 3490.0 MPa (45° double-plate construct), 1754.0 MPa (90° double-plate construct), 1123.0 MPa (135° double-plate construct), and 816.5 MPa (180° double-plate construct). The additional short plate dispersed some stress leading to the decrease in the peak VMS of the lateral plate. As angle formed by the double plates increased, the dispersed function of the additional plate was becoming obvious. The bending angles of the lateral plate were 18° versus 12° versus 3° versus 2° versus 1° (the single-plate construct versus 45° double-plate construct versus 90° double-plate construct versus 135° double-plate construct versus 180° double-plate construct).

**Conclusions:**

Our study indicated that increasing the angle between the plates in a double-plate construct improves the stability of the construct over a single lateral plate when there is no cortical support opposite to the lateral plate. The strongest fixation occurred when the angle between the two plates was greater than ninety degrees.

## 1. Introduction

Femoral shaft nonunion is relatively common with a rate between 1% and 20% depending on the type of fracture and on the technique used [1–5]. Various treatment strategies, including nail dynamization, Ilizarov technique, nail exchange, locking plate osteosynthesis, and the combinations thereof, are available to treat the femoral shaft nonunions and get the success rate ranging between 47 and 100% [2, 6–8]. Despite the success rate reported in the literature for these revision techniques, each modality has its own set of problems. Nail dynamization is suggested for patients without segmental bony defects and suffering from the main complication of bone shortening that can lead to significant leg length discrepancy. And highly comminuted and oblique fractures have a high risk of developing significant shortening [9]. Ilizarov external fixation often requires prolonged use that leads to the issues of pin tract infections, living inconvenience, and discomfort [10, 11]. Study has reported a 78.3% successful rate of exchange reamed femoral nailing [12]. And reaming operation in the exchange nailing surgery causes considerable vascular damage to the blood circulation of the endosteum [9, 13]. Plate osteosynthesis can provide the constant fracture compression as well as a chance of sufficient debridement to remove the fibrous scar, which is also favored by surgeons with the union rates of 96% [3, 7, 8, 14–16].

The AO/OTA 32-B3 C1 and C2 fracture types were characterized by the medial cortex of the femoral diaphysis fragmented so that it provided no construct stability. A single plate placed laterally in this fracture types is subjected to a local concentration of bending forces which may induce failure [17]. Thus, techniques of double-plate fixation have been emerging as a viable fixation scenario for managing the femoral shaft nonunion model that is plating without a competent cortex opposite the plate [18–20]. But, to the best of our knowledge, there are not any mechanical studies of finite element analysis test being published to compare the mechanical stability of the different double-plate angles as fixation methods used in the femoral shaft nonunion with no medial support opposite the primary lateral plate. Therefore, in an effort to shed more light on this issue, we designed this research to evaluate the mechanical strength of the different double-plate fixation patterns in the treatment of the femoral shaft nonunion without medial support. We hypothesized that the greater the angle between the two plates is, the stronger the construct would be.

## 2. Materials and Methods

**Figure 1:**
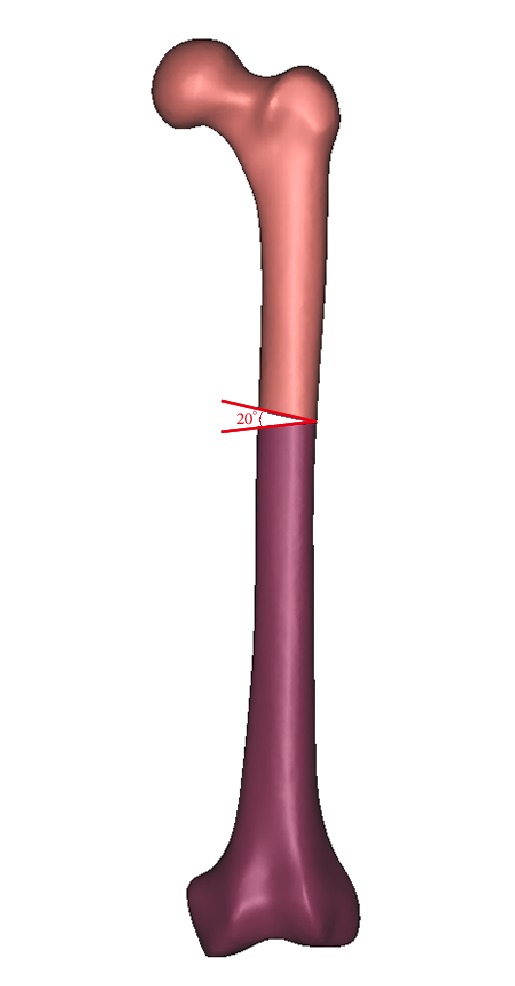
A wedge fragment of which proximal and distal simulated line formed an angle of 20° was created and removed, and finally we got the model.

The geometric model of femur was employed from a three-dimensional model of a left fourth-generation composite femur (MODEL3405#, Pacific Research Laboratories, Vashon, WA). Then we constructed the fracture model in the 3-matic (Materialize, Belgian) to simulate the model of femoral shaft nonunion with no cortical support opposite the primary lateral plate (Figure 1). A wedge fragment of which proximal and distal simulated line formed an angle of 20° was created and removed, and we finally got the model. There was totally no contact between the proximal and distal fragments.

**Figure 2:**
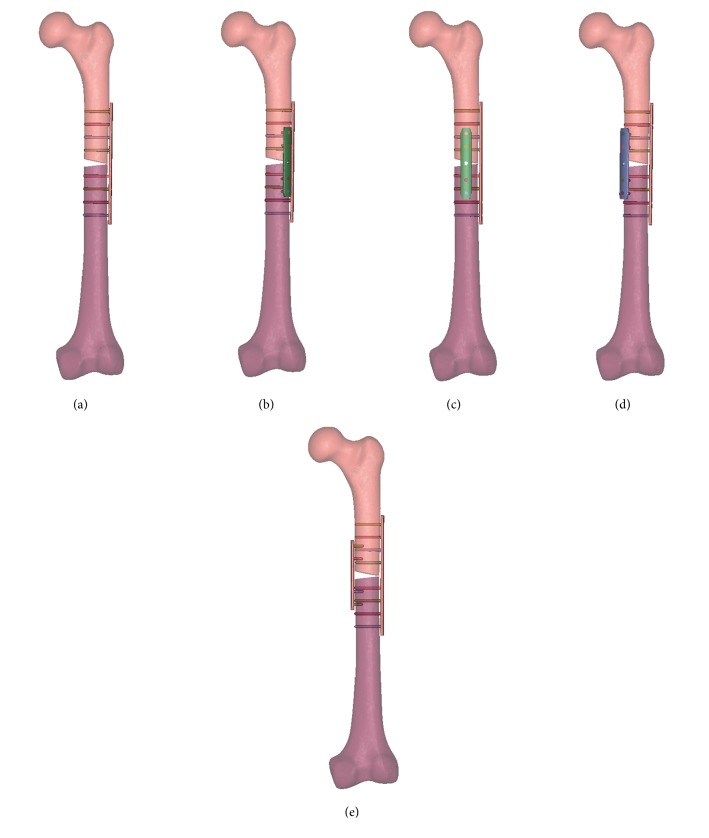
Assemblage of the internal fixations and bones was accomplished in 3-matic to simulate the single-plate construct (a), 45° double-plate construct (b), 90° double-plate construct (c), 135° double-plate construct (d), and 180° double-plate construct (e).

According to the manufacturer-provided engineering drawing, we reconstructed the geometric 3D models of lateral plate, additional plate, and screws using the computer aided design (CAD) software of Unigraphics NX 8.5 (Siemens PLM Software). The thread screws were replaced by smooth surfaces, the size of which was corresponding to the average diameter of the given thread. Assemblage of the internal fixations and bones was accomplished in 3-matic to simulate the single-plate construct, 45° double-plate construct, 90° double-plate construct, 135° double-plate construct, and 180° double-plate construct (Figure 2). All the models were meshed using the software HyperMesh 11.0 (Altair Engineering, Inc, USA).

The assembled 3D models were then imported into ABAQUS (Simulia, France) to generate the finite element models. The synthetic bone was assumed to be homogeneous, isotropic with linear elastic properties as reported by the manufacturer and previous studies [21–23]. The modulus of elasticity and Poisson's ratio of the bone were shown in Table 1. The plates and screws were made of titanium alloy (Table 1). Tetrahedral 10-node elements (C3D10) were applied to the finite element models. The effect of gravity was considered as negligible in the model.

Frictional contact interactions were assumed between the different parts of the models. There were not any contact between fragments. The interfaces between bone and internal fixations were simulated by contact pairs with a friction factor of 0.3 [24]. The interface of screws and locking plate was set as tie to simulate the locking condition. All nodes on the surface of distal femur were constrained with 0 degrees of freedom to prevent rigid body motions during the analysis. The FE models were applied a load of 800N corresponding to 100% body weight and the force was introduced to the center of the femoral head. The force vector was pointing laterally in the coronal plane at an angle of 13° with the axis of the femoral shaft and posteriorly at an angle of 8° with the shaft axis in the sagittal plane [25].

Convergence tests were performed in the single-plate construct model to ensure a sufficiently fine element discretization. The displacement at the point of load application in the direction of the load was computed as a function of the total number of elements.

Three parameters were used to capture the mechanical factors involved in the fixation stability and fracture healing. They included the von Mises Stress (VMS) distribution of the implant, peak VMS, and model displacement and deformation.

## 3. Results

The number of elements in the nodes and models are shown in the Table 2. And more details about mesh size were listed in Table 3.

**Figure 3:**
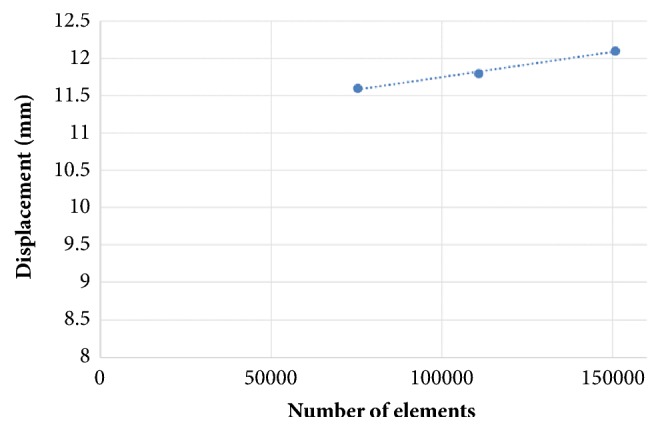
Results of the mesh convergence study. Displacement at the point of load application in a single-plate construct model as a function of the number of elements.

The mesh convergence study showed that the increase in displacement was <2% when the number of femur elements increased from 75346 to 150766. Numbers of elements more than 150000 were used as the optimal mesh size and resolution (Figure 3).

### 3.1. von Mises Stress (VMS) Distribution

**Figure 4:**
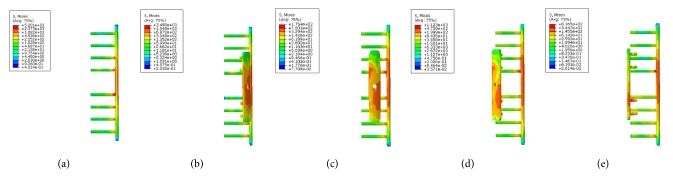
VMS distribution in five fixation systems: (a) the single-plate construct. (b) 45° double-plate construct. (c) 90° double-plate construct. (d) 135° double-plate construct. (e) 180° double-plate construct. Stresses appeared to be concentrated in middle surface of the lateral plate near the fragment of each group. Highest stress values were observed in the two locking screws closest to the fracture line.

**Figure 5:**
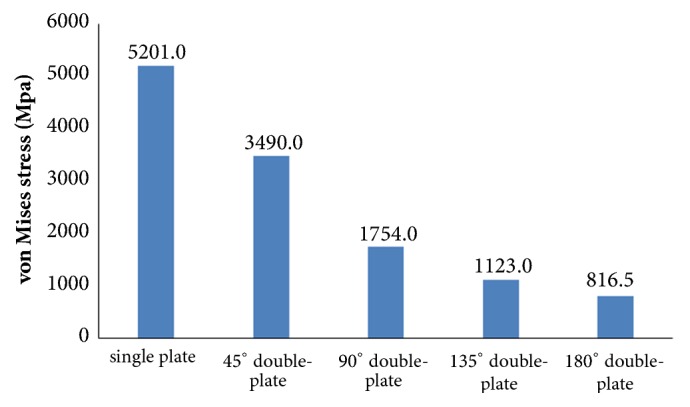
Graphic demonstration of the peak VMS in five fixation systems.

**Figure 6:**
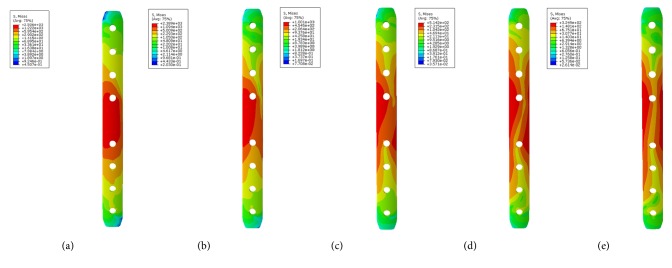
VMS distribution in lateral plate: (a) the single-plate construct. (b) Lateral plate of 45° double-plate construct. (c) Lateral plate of 90° double-plate construct. (d) Lateral plate of 135° double-plate construct. (e) Lateral plate of 180° double-plate construct.

**Figure 7:**
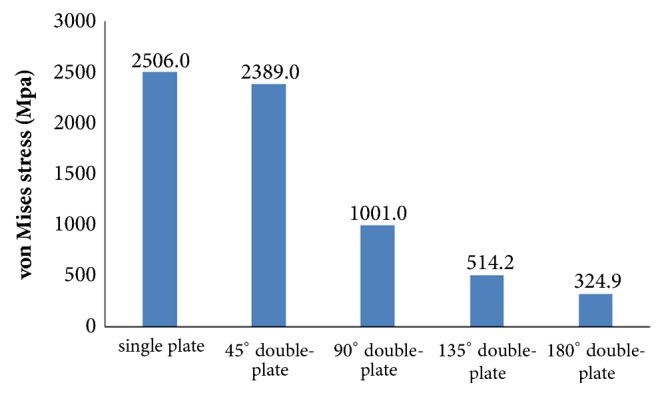
Graphic demonstration of the peak VMS in lateral plate.

**Figure 8:**
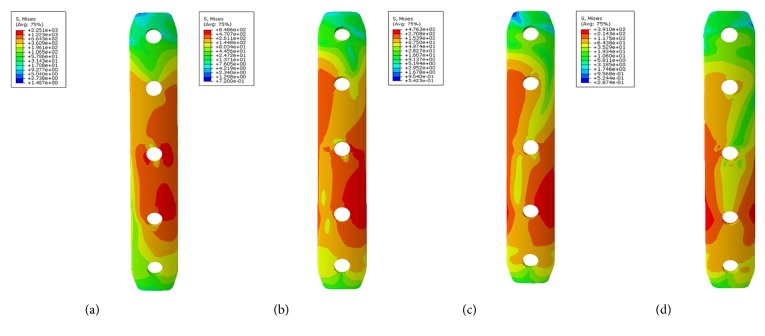
VMS distribution in the additional plate. (a) Additional plate of 45° double-plate construct. (b) Additional plate of 90° double-plate construct. (c) Additional plate of 135° double-plate construct. (d) Additional plate of 180° double-plate construct.

**Figure 9:**
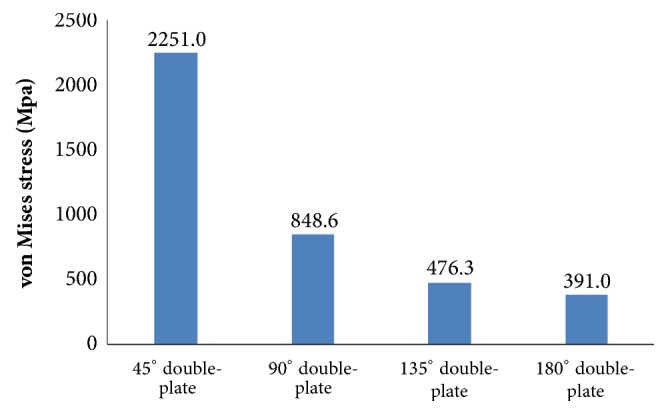
Graphic demonstration of the peak VMS in the additional plate.

Large differences in the stress distribution were observed on the five fixation systems. Peak stress value of the implants was highest in the single-plate model. As to the double-plate models, 45° double-plate construct model showed the highest stress values on the implant. And the lowest stress values were observed in the 180° double-plate model. Stresses appeared to be concentrated in middle surface of the lateral plate near the fragment of each group (Figures 4 and 5). The additional short plate dispersed some stress leading to the decrease in the peak VMS of the lateral plate. As the angle formed by the second plate increased, the function of the additional plate became obvious as the forces were lessened (Figures 6–9).

### 3.2. Model Displacement and Deformation

**Figure 10:**
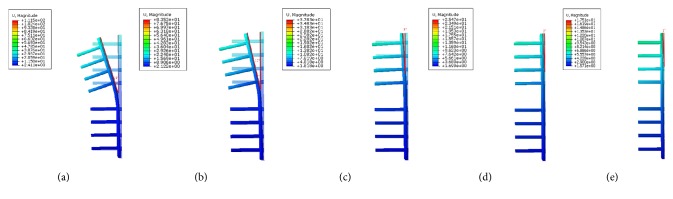
The displacement nephogram demonstration of model displacement and deformation. (a) The single-plate construct with deformation angle of 18°. (b) Lateral plate of 45° double-plate construct with deformation angle of 12°. (c) Lateral plate of 90° double-plate construct with deformation angle of 3°. (d) Lateral plate of 135° double-plate construct with deformation angle of 2°. (e) Lateral plate of 180° double-plate construct with deformation angle of 1°.

**Figure 11:**
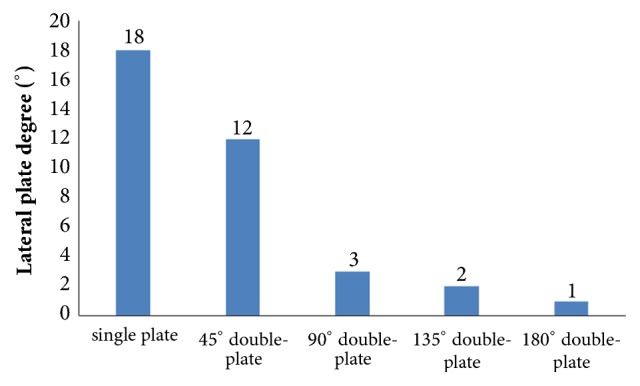
Graphic demonstration of the deformation angle in five fixation constructs.

The displacement nephogram demonstrated that values of model displacement in the double-plate systems were lower than the single-plate model. As the angle formed by the second plate increased, the angle of deformation decreased. The single-plate construct represented the largest deformation as compared to other models which is depicted by the measured angles (Figures 10 and 11). On the other hand 180° double-plate model with the lowest amount of deformation remained the most stable fixation choice.

## 4. Discussion

In the present study, we investigated whether the different angles formed by the double locking plates had the mechanical distinction in the treatment of femoral shaft nonunion with no cortical support opposite the primary lateral plate. To clarify this problem, we adopted this finite element analysis research that showed the different angles fixation scenarios affected the fixation stability. This study demonstrated that the greater the angle between the two plates is, the more stable the fixation construct became. The double-plate fixation technique may be an effective and reliable alternative in treating femoral nonunion when considering the treatment principle of solid fixation and good bone contact at the nonunion site [26, 27].

Femoral shaft nonunion with no cortical medial support treated with a single laterally locking plate is subjected to a local concentration of bending forces and fails to provide cortical support medially, which was proved by the finite element results in this study. Thus, the double-plate fixation is emerging as a reasonable alternative to treat the nonunion. A union rate above 90% has been reported in studies [18–20], but double-plate fixation principle has never been proposed. In 2015, Maimaitiyiming et al. [18] reported 14 consecutive patients with femoral shaft aseptic nonunion were treated with double-plate fixation combined with bone grafting and all cases achieved bony union in the average time of 5.2 months. This study suggests that implants should be placed on the lateral and anterior sides, at a 90° angle with each other. In 2016, Peng et al. [20] demonstrated that double locking plates fixation could serve as an effective therapy strategy for femoral shaft nonunion. Jiang et al. [19] got the union rate of 100% by using the fibular autograft with double locking plates for the treatment of diaphyseal femoral nonunion. However the number of cases in the literature is so few that recommendation of the double-plate technique could not be proposed. The mechanical results of this study provide some guidance to its application in clinical surgery. As shown in Figure 10, under the axial loading, the 180° double-plate construct provided more stable fixation than the other models and the deformation angles showed in Figure 11 indicated that stability was increasing as the angles between the double-plate increased. Figures 8 and 9 illustrated the additional plate dispersed some stress and the quantity of the dispersed stress was changed as the angle increased. The peak VMS on the fixation construct decreased as the angle formed by the second plate increased, indicating the function of the additional plate became obvious (Figures 5 and 6). The results mentioned above indicated that a double-plate angle construct larger than 90° provided better stability by allowing the transmission of the body weight through the axis of the femoral shaft, resulting in the lower stress distribution on the plate and greater security of the fixation system. Double plates enabled the fixation construct to generate a rigid, stable mechanism and to provide a strong resistance ability to counter shear effect.

No experimental validation was conducted, which clearly is a limitation. However, our aim was to examine trends rather than absolute values and to show that double plates of more than 90° are less prone to failure. In this respect, the lack of experimental validation is justified. A previous experimentally validated numerical study [21–25] employed the same loading and boundary conditions as our study. And this study investigated the fixation stability without calculating the intrafragmentary motion at the fracture site. Despite these limitations, this study is the first finite element analysis study comparing the mechanical efficiency of five clinically used double-plate configurations (single locking plate, 45° double-plate, 90° double-plate, 135° double-plate, and 180° double-plate) in femoral shaft nonunion with no cortical support opposite the primary lateral plate. In this study, some important aspects should be paid attention to: (1) the numbers of the locking screws in every models were the same. The lateral plate is the main plate which is long and with 8 bicortical locking screws. The medial plate is shorter with 4 unicortical locking screws. (2) All screws were added to the plate using the assemble function in the UG software, because screws can run along the center line of every hole on the plate.

We have described the finite element analysis of increasing the angle between double locking plates in a model of a bending wedge fracture nonunion where there is lack of medial cortical support. As angle between the two plates increased, stability increased and less stress distribution occurred when the angle between the double plates was more than 90°. This finite element simulation may facilitate the further mechanical research and help guide the treatment of the femoral shaft nonunion clinically.

## 5. Conclusions

In conclusion, our study indicated that increasing the angle between the plates in a double-plate construct improves the stability of the construct over a single lateral plate when there is no cortical support opposite to the lateral plate. The strongest fixation occurred when the angle between the two plates was greater than ninety degrees. This study will require clinical confirmation as to its practicality in the management of nonunions with defects opposite the stabilizing plate.

## Figures and Tables

**Table 1:** Material properties used in the current study (Ti-6AL-4V, cortical, and trabecular bone).

Ti-6AL-4V		Bone			
		cortical		trabecular	
E (GPa)	Poisson's ratio	E (GPa)	Poisson's ratio	E (GPa)	Poisson's ratio

105	0.35	16.7	0.26	0.155	0.3

**Table 2:** The elements and nodes of the models in this study.

		Single plate	45° double- plate	90° double- plate	135° double- plate	180° double- plate
Lateral plate (with screws)	Elements	90368	90368	90368	90368	90368
	Nodes	144077	144077	144077	144077	144077

Added plate (with screws)	Elements	0	23108	23108	23108	23108
	Nodes	0	37713	37713	37713	37713

Femur	Elements	150766	174926	168795	165117	159241
	Nodes	29858	34211	33106	32489	31510

**Table 3:** The mesh size of the models in this study.

Models	Mesh size (mm)
Lateral plate	1.3
The screw of lateral plate	0.7
Added plate	1.3
The screw of added plate	0.8
Femur	Maximum: 3; minimum: 1

## Data Availability

The data used to support the findings of this study are available from the corresponding author upon request.
